# Epstein-Barr virus lytic gene expression is tightly linked to ER stress but not cytotoxicity with bortezomib or nelfinavir

**DOI:** 10.1186/1750-9378-7-S1-P32

**Published:** 2012-04-19

**Authors:** Courtney M Shirley, Nene Kalu, Meir Shamay, Richard F Ambinder

**Affiliations:** 1 Department of Oncology, Johns Hopkins University School of Medicine, Baltimore, MD, USA

## 

Epstein-Barr virus (EBV) is associated with AIDS-related lymphomas and other malignancies. We have previously shown that the proteasome inhibitor bortezomib is an activator of EBV lytic gene expression and that these effects are mediated by ER stress and the unfolded protein response (UPR) [1]. We investigated the relationship between the induction of UPR and EBV lytic gene expression with a variety of UPR inducers, as well as the association of their viral and antitumor effects in a variety of tumor cell lines. Bortezomib, thapsigargin, and tunicamycin activate the UPR and EBV lytic cycle. Recently, nelfinavir has also been reported to lead to ER-stress and the UPR. We found a dose-dependent relationship with bortezomib and with nelfinavir for the induction of the UPR markers (Bip, ATF4, XBP1s, and CHOP10), EBV lytic gene expression (measured as ZTA RNA), and cell toxicity in Burkitt’s lymphoma cell lines.. Blocking ER stress and UPR activation, by cycloheximide (CHX) treatment or by Bip knockdown, diminished ZTA induction but had no effect on cellular toxicity. We also studied EBV lymphoblastoid cell lines (LCLs). In contrast to the BL cell lines, bortezomib did not induce ER stress, activate the UPR or lead to EBV lytic gene expression but was nonetheless toxic to LCLs. These results indicate that bortezomib and nelfinavir both induce ER stress and UPR leading to EBV lytic reactivation in BL cells. UPR induction corresponds with EBV lytic gene induction but appears to be distinct from cellular toxicity. Our findings suggest that ER stress, UPR and viral activation are closely linked but may be separable from the cytotoxic effects of some pharmacologic inducers. Bortezomib and nelfinavir may serve as laboratory and clinical tools for manipulating viral gene expression in EBV associated malignancies.
